# Fecal Metabolomic Insights into Memory-Associated Pathways Modulated by *Bacopa monnieri*, Mixed Thai Berry, and Combined Extracts in Rats Under Chronic Unpredictable Mild Stress

**DOI:** 10.3390/antiox15010056

**Published:** 2026-01-01

**Authors:** Kalyarut Phumlek, Nitra Nuengchamnong, Phichsinee Rerkshanandana, Sutisa Nudmamud-Thanoi, Worawut Chaiyasaeng, Nathareen Chaiwangrach, Wiyada Khangkhachit, Plaiyfah Janthueng, Wanfrutkon Waehama, Kornkanok Ingkaninan, Prapapan Temkitthawon

**Affiliations:** 1 NU Pilot Plant for Herbal Extracts and Products, Center of Excellence for Natural Health Product Innovation, Faculty of Pharmaceutical Sciences, Naresuan University, Phitsanulok 65000, Thailand; kalyarut.ph@nu.ac.th (K.P.); phichsineer65@nu.ac.th (P.R.); worawut.ch@nu.ac.th (W.C.); nathareenc61@nu.ac.th (N.C.); wiyada.kh@nu.ac.th (W.K.); 2 Research and Innovation Cluster for Natural Health Products, Faculty of Pharmaceutical Sciences, Naresuan University, Phitsanulok 65000, Thailand; 3 Science Laboratory Centre, Faculty of Science, Naresuan University, Phitsanulok 65000, Thailand; nitran@nu.ac.th; 4 Department of Anatomy, Centre of Excellence in Medical Biotechnology, Faculty of Medical Science, Naresuan University, Phitsanulok 65000, Thailand; sutisat@nu.ac.th (S.N.-T.); plaiyfahj66@nu.ac.th (P.J.); wanfrutkonw65@nu.ac.th (W.W.)

**Keywords:** *Bacopa monnieri*, mixed berry, fecal metabolomics, cognition, chronic stress

## Abstract

Chronic stress impairs cognition through gut–brain axis dysregulation and metabolic imbalance. This study applied untargeted fecal metabolomics to investigate the cognitive and metabolic effects of *Bacopa monnieri* (L.) Wettst (Brahmi), mixed Thai berry, and their combined extracts in rats exposed to chronic unpredictable mild stress. Cognitive performance was evaluated using the novel object recognition test. Fecal metabolites were profiled using LC-ESI-QTOF-MS, followed by data preprocessing and multivariate statistical analysis. Orthogonal partial least squares regression was applied to identify metabolites associated with the recognition index, and pathway enrichment analysis was subsequently performed to interpret biological relevance. All interventions were associated with improved recognition performance and treatment-related metabolic modulation. Biosynthesis of unsaturated fatty acids was consistently enriched across treatment groups, indicating a shared involvement of lipid remodeling. Treatment-specific responses were also observed: Brahmi was associated with linoleic and alpha-linolenic acid metabolism; mixed Thai berry extract demonstrated dose-dependent modulation of lipid metabolism, with low-dose supplementation additionally yielding amino-acid-derived metabolites; and bile acid-related signaling was uniquely detected in the low-dose combined extract group. These findings demonstrate that fecal metabolomics can capture distinct metabolic signatures associated with herbal extract-induced cognitive improvement and highlight lipid remodeling as a shared metabolic feature across interventions under chronic stress.

## 1. Introduction

Cognitive impairment and affective disorders arising from chronic stress are increasingly recognized as global health challenges, adversely affecting quality of life and increasing risk for neurodegenerative diseases [1,2]. The mechanisms underlying stress-induced cognitive decline involve intricate bidirectional communication between the gut and the brain, known as the gut–brain axis. This network integrates neural, endocrine, immune, and metabolic signals, with gut microbiota and their metabolites playing pivotal modulatory roles in neurocognitive outcomes [3].

Recent advances in metabolomics have facilitated untargeted profiling of fecal metabolites, allowing the investigation of gut microbiota–host interactions at the molecular level [4]. Fecal metabolomic signatures reflect gut ecosystem adaptation, dietary intake, and systemic physiological changes, and have emerged as potential indicators of neurobehavioral outcomes under stress [5]. Notably, metabolic disturbances in lipid, amino acid, and bile acid pathways can influence neurotransmission, neuroinflammation, and brain energy metabolism, which are key processes implicated in stress resilience and cognitive function [5,6].

Herbal interventions are increasingly recognized for their potential to modulate cognitive function, with particular interest in *Bacopa monnieri* (L.) Wettst. (Brahmi) and mixed Thai berry extracts due to their antioxidant and neuroprotective properties.

Brahmi, a traditional medicinal herb used in Thai traditional and Ayurvedic medicine, has gained considerable scientific attention as a neuroactive botanical owing to its diverse phytochemical profile and reported pharmacological activities. Brahmi is rich in saponin glycosides and polyphenolic constituents, which contribute to its antioxidant capacity [7] and its ability to mitigate oxidative stress-related damage in neural tissue [8]. Beyond antioxidative effects, Brahmi has demonstrated notable anti-inflammatory actions, including suppression of neuroinflammatory signaling cascades that are implicated in the progression of cognitive decline and neurodegeneration [9]. Emerging multiomics and mechanistic studies further suggest that Brahmi interacts with pathways governing neuroplasticity, cytokine signaling, and extracellular matrix regulation, thereby supporting neuronal resilience under pathological conditions such as Alzheimer’s disease [10,11]. Complementing these mechanistic insights, both controlled clinical trials and behavioral studies have reported improvements in memory performance, attention, and learning capacity across diverse populations, including children and adults [12,13].

Mixed Thai berry, including *Morus alba* L. (mulberry), *Antidesma ghaesembilla* Gaertn. (mamao), and *Syzygium nervosum* DC. (making), are rich in polyphenols, flavonoids, and anthocyanins [14,15,16], which contribute to their antioxidant [14,16], and neuroprotective properties [17,18,19]. Clinical and preclinical studies have increasingly demonstrated the beneficial effects of regular mixed berry consumption on cognitive function and metabolic health. Nilsson et al. (2017) reported that daily intake of a mixed berry beverage significantly improved cognitive performance and reduced cardiometabolic risk markers in healthy older adults [20]. In agreement, Huang et al. (2023) demonstrated that supplementation with a mixture of Nordic berries improved spatial memory and modulated gut microbiota composition in C57Bl/6J mice, including increasing the abundance of *Akkermansia muciniphila*, a microbial taxon associated with favorable cognitive outcomes [21]. Beyond direct antioxidant actions, berry polyphenols may exert additional neurobiological effects through interactions with the gut microbiota. Dietary polyphenols undergo extensive microbial metabolism, generating a range of smaller phenolic metabolites that can be absorbed systemically and are proposed to contribute to brain-related outcomes via gut–brain axis signaling [22,23]. In this context, a mixed Thai berry formulation may reasonably be considered to have health-promoting potential, based on documented antioxidant, metabolic, and neuroprotective actions of its constituent fruits and evidence from broader mixed berry consumption studies.

Although Brahmi has demonstrated cognitive benefits, its characteristic bitterness presents challenges for routine consumption and product development. Combining Brahmi with mixed Thai berry extract may improve palatability and enhance acceptability, allowing the combined extracts to be considered in practical contexts such as functional foods or nutraceutical formulations. Evaluating the metabolic and cognitive profiles of such combined extracts is therefore relevant not only from a mechanistic perspective but also in the context of potential real-world applications.

The chronic unpredictable mild stress (CUMS) paradigm in rodents is widely employed to model prolonged psychological stress in humans, as it induces behavioral deficits, alterations in gut microbiota composition, and metabolic disturbances associated with cognitive dysfunction. Recent findings indicate that CUMS exposure leads to perturbations in several critical metabolic pathways, including glycerolipid metabolism, fatty acid oxidation, tryptophan catabolism, and bile acid homeostasis, each of which is implicated in neural signaling, synaptic plasticity and memory processes [24]. Metabolomic profiling has therefore emerged as a valuable approach for characterizing biochemical alterations associated with stress-related behavioral outcomes.

Although accumulating evidence supports the cognitive benefits of Brahmi and mixed berry extracts, the metabolic pathways underlying these effects under chronic stress remain poorly defined. Additionally, whether the biological impact differs between single-extract and combined supplementation, and whether mixed berry intake exhibits dose-dependent effects, has not been systematically evaluated. Fecal metabolomics provides an appropriate platform to investigate these questions, as fecal metabolites reflect host physiology, dietary metabolism, and microbially derived biotransformation products.

To address these gaps, the present study employed untargeted fecal metabolomics in a CUMS rat model receiving a single dose of Brahmi extract, low- or high-dose mixed Thai berry extract, or low- or high-dose combined extract. Metabolite features were analyzed in relation to recognition memory performance, and pathway enrichment analysis was conducted to identify treatment-associated metabolic pathways affected under chronic stress. This approach enables evaluation of dose and combination effects at the metabolic level and provides insight into the biochemical signatures associated with recognition memory following herbal intervention.

## 2. Materials and Methods

### 2.1. Brahmi Extract, Mixed Thai Berry Extract, and Combined Extract (BmixB) Preparation

Aerial parts of *B. monnieri* (Plantaginaceae), commonly known as Brahmi, were obtained from Thap Yai Chiang, Phitsanulok Province, Thailand. The dried and powdered materials were extracted with 95% ethanol and concentrated under reduced pressure, yielding a dried extract. Prior to use in animal studies at the specified dose, the Brahmi extract was standardized to contain 5.0% *w*/*w* total saponin glycosides, determined based on the combined quantification of jujubogenin glycosides (bacoside A3 and bacopaside X) and pseudojujubogenin glycosides (bacopaside I, bacopaside II, and bacopasaponin C).

Ripe fruits of *M. alba* (Moraceae), *A. ghaesembilla* (Phyllanthaceae), and *S. nervosum* (Myrtaceae) were sourced from certified growers in Nan Province, Thailand. The ripe fruits were extracted with 5% acetic acid in 95% ethanol. The filtrate was concentrated under reduced pressure at 40 °C and kept at −20 °C until use. The mixed berry extract showed a total anthocyanin content of 41.70 ± 1.66 mg cyanidin-3-O-glucoside equivalents (C3GE) per gram of extract, determined using the pH differential method [25].

For the BmixB extract, the standardized Brahmi and mixed berry extracts were blended at a fixed ratio corresponding to the dose calculated for animal study. The ratio was determined based on the concentrations of their respective bioactive markers, total saponin glycosides in the Brahmi extract and total anthocyanins in mixed Thai berry extract.

### 2.2. Animals and Experimental Design

All experiments were conducted in accordance with institutional guidelines, under approval from the Naresuan University Animal Care and Use Committee (NUACUC, protocol NU-AE660305). Eighty-four male Sprague Dawley rats (7 weeks old) were obtained from Nomura Siam International Co., Ltd. Animals were housed in a controlled environment at 22 ± 1 °C, with a 12 h light/12 h dark cycle and relative humidity maintained at 55 ± 10%. Standard chow and water were provided ad libitum. A one-week acclimatization period preceded random assignment of animals to the experimental groups.

#### 2.2.1. Chronic Unpredictable Mild Stress Procedure

Stress induction was performed using the CUMS protocol, a widely used paradigm for modeling chronic psychological stress in rodents. Over a period of 14 consecutive days, animals were exposed each day to a predetermined and varied sequence of mild stressors. These included wet sawdust, food deprivation with lights off, removal of sawdust, water deprivation with lights off, cage tilting at 45°, forced swimming, and restraint. Most stressors were applied during the dark phase, except for forced swimming and restraint, which were performed during the light phase. The order of stressor application was systematically rotated throughout the experimental period to maintain unpredictability of chronic stress and minimize habituation. A 14-day CUMS duration was selected based on previous studies demonstrating that two-week exposure is sufficient to induce cognitive and behavioral alterations in rats [26,27].

#### 2.2.2. Treatment Administration

Rats (*n* = 12 per group) were randomly assigned to 7 experimental groups: Control-vehicle (unstressed control group receiving vehicle, Ctl), CUMS-vehicle (stressed control group subjected to CUMS protocol and receiving vehicle, CUMS), CUMS-Brahmi (CUMS-exposed group receiving Brahmi extract at 20 mg/kg BW), CUMS-Berry Low (CUMS-exposed group receiving low-dose mixed berry extract containing 0.043 mg C3GE/kg BW), CUMS-Berry High (CUMS-exposed group receiving high-dose mixed Thai berry extract containing 0.215 mg C3GE/kg BW), CUMS-BmixB Low (CUMS-exposed group receiving a combination of Brahmi extract at 20 mg/kg BW and low-dose mixed Thai berry extract), and CUMS-BmixB High (CUMS-exposed group receiving a combination of Brahmi extract at 20 mg/kg BW and high-dose mixed Thai berry extract). All treatments were administered orally once daily for 14 days, and body weight was recorded daily before dosing.

The Brahmi dose of 20 mg/kg BW was selected based on Uabundit et al. (2010) [28], which demonstrated cognitive and neuroprotective effects in rodent models; therefore, this dose was adopted for the present study. For the mixed Thai berry extract, the low and high doses were derived from Kim and Oh (2012) [29], where significant cognitive effects were observed at 100 and 500 mg/kg BW. Based on the reported anthocyanin content (0.43 ± 0.02 mg C3GE/g extract), the equivalent doses corresponded to 0.043 and 0.215 mg C3GE/kg BW and were used to examine potential dose–response effects.

#### 2.2.3. Novel Object Recognition (NOR) Test

Recognition memory performance was assessed using the NOR test. The arena was illuminated under standard laboratory lighting conditions, and neutral wooden geometric blocks of similar dimensions were used as objects. Rats were habituated to the open-field arena (76 cm × 76 cm × 42 cm) for 10 min. During the familiarization phase, two identical objects were placed in the arena, and each rat was allowed to explore them for 5 min. After a 90 min retention interval, one of the familiar objects was replaced with a novel object for a 5 min test trial. Exploration was defined as head-oriented contact within 3 cm of an object and was quantified using SMART 3.0 video-tracking software. Increased investigation of the novel object was used as an index of recognition memory. The recognition index (RI) was calculated as the time spent investigating the novel object divided by the total time spent exploring both the novel and familiar objects [30].

#### 2.2.4. Feces Sample Collection

Fecal samples used for metabolomics analysis were collected on day 14 of the experimental period. Fresh fecal samples were collected directly into sterile tubes and immediately stored at −80 °C until extraction and analysis.

### 2.3. Feces Sample Preparation for Metabolic Profiling

The sample preparation protocol was adapted from Minale et al. (2023) [13]. Fecal samples were dried at 40 °C using a SpeedVac concentrator (Labconco Corporation, Kansas City, MO, USA). Ten mg of dried feces were extracted in 1 mL of cold 80% methanol containing 0.1% formic acid. The mixture was vortexed for 2 min and sonicated for 10 min to enhance metabolite extraction, then kept at −20 °C for 2 h. After centrifugation at 14,000 rpm at 4 °C for 20 min, the supernatant was collected and concentrated to dryness using a SpeedVac concentrator. The dried residue was reconstituted in methanol and adjusted to a final concentration of 20 mg/mL. All prepared samples were transferred to HPLC vials and stored at −20 °C before LC-MS analysis. Quality control (QC) samples were prepared by pooling equal aliquots (10 μL) from each extract. Blank samples were analyzed periodically to assess potential contamination and carryover under the LC-MS conditions.

### 2.4. LC-ESI-QTOF-MS Analysis Conditions

Metabolite profiling was conducted using an Agilent 1290 Infinity II LC system interfaced with an Agilent 6575A Revident Quadrupole Time-of-Flight (QTOF) mass spectrometer (Agilent Technologies, Santa Clara, CA, USA). Chromatographic separation was achieved on a ZORBAX Eclipse Plus C18 column (3.0 mm × 100 mm, 1.8 μm; Agilent Technologies) equipped with a corresponding guard column (3.0 mm × 5 mm, 1.8 μm). The chromatographic run employed a binary solvent system consisting of (A) 0.1% formic acid (*v*/*v*) in water and (B) 0.1% formic acid (*v*/*v*) in acetonitrile. The gradient elution program started at 95% A and 5% B, linearly decreased to 5% A and 95% B within 20 min, and was maintained for 5 min, followed by a 5 min post-run re-equilibration. The flow rate was set at 0.5 mL/min, the column temperature at 35 °C, and the injection volume at 5 μL.

Mass spectrometric detection was performed using a Dual AJS electrospray ionization (ESI) source operated in positive polarity. The optimized source and ion optical parameters were as follows: drying gas (nitrogen) flow rate 8 L/min; gas temperature 325 °C; sheath gas temperature 250 °C with flow rate 11 L/min; nebulizer pressure 30 psi; capillary voltage 3500 V; nozzle voltage 1000 V; fragmentor potential 175 V; skimmer voltage 45 V; and octopole RF voltage 750 V. Full-scan data were acquired over the *m*/*z* range 100–1000. Prior to sample analysis, the QTOF instrument was calibrated using an LC/MS ESI-X tuning mix (Agilent Part No. 5191-6449) to ensure mass accuracy and peak shape, and calibration was performed at the beginning of each analytical batch. Continuous reference mass correction was applied in positive mode using ions at *m*/*z* 121.0508 and 922.0097. Total ion chromatograms (TIC) were recorded in profile mode, whereas full-scan MS data were acquired in centroid mode.

### 2.5. Data Analysis

Metabolomics data analysis was performed following a structured workflow integrating data acquisition, preprocessing, statistical analysis, and pathway interpretation. Raw LC-ESI-QTOF-MS spectral data were collected from fecal samples using MassHunter software (Data Acquisition v12.1.98.0 and Qualitative Analysis v12.0; Agilent Technologies) and converted to mzML format using MSConvert software version 3.0 (ProteoWizard). Data pre-processing was conducted in MZmine 4.3.0, including filtering, peak detection, deconvolution, alignment, and normalization steps.

Untargeted data processing in MZmine yielded 4166 aligned metabolic features. Procedural blanks were used to identify background-derived signals, and any feature detected in blank runs was removed using a presence/absence criterion. Peak-based gap filling (Peak Finder algorithm in MZmine) was applied prior to CV filtering, after which 1859 features remained. Metabolic features were further quality-filtered based on the coefficient of variation (CV), retaining features with CV < 30% as assessed by MetaboAnalyst 6.0; 1056 features met this threshold. The median CV of pooled QC samples was 19.24%. Feature intensities were log-transformed and Pareto-scaled prior to multivariate analysis.

To evaluate data quality and sample consistency, Principal Component Analysis (PCA) was initially performed across all and QC samples. The resulting PCA score plot enabled visualization of sample clustering, the detection of batch effects, and assessment of overall data reproducibility prior to downstream analysis.

Initial group differences between the Ctl and CUMS groups were explored by unsupervised PCA and subsequently confirmed by supervised Orthogonal Partial Least Squares-Discriminant Analysis (OPLS-DA) using SIMCA-P 13.0.3.0 (Umetrics, Umeå, Sweden). The OPLS-DA model enabled clear discrimination of metabolic profiles between the two groups, validating the impact of chronic unpredictable mild stress at the metabolome level.

For treatment effects, pairwise OPLS regression (OPLS-R) analyses were performed to assess metabolic differences between the CUMS group and each treatment group, specifically: CUMS versus (vs.) CUMS-Brahmi, CUMS vs. CUMS-Berry Low, CUMS vs. CUMS-Berry High, CUMS vs. CUMS-BmixB Low, and CUMS vs. CUMS-BmixB High. This approach enabled the identification of metabolites and metabolic pathways most strongly associated with the effects of each intervention relative to chronic stress. Candidate metabolites were ranked according to positive OPLS model coefficients. Only metabolites with positive coefficients were retained, as the analysis focused on those associated with improved recognition performance; metabolites with negative coefficients were excluded. The top 30 metabolites with the highest coefficient values and VIP > 1.0 were selected as key activity-related markers for downstream pathway enrichment analysis. Putative metabolite identification was performed by matching measured *m*/*z* and retention time (RT) values against entries in the MassHunter METLIN Metabolite Personal Compound Database and Library (PCDL) (Agilent Technologies), and by matching measured *m*/*z* values against the Human Metabolome Database (HMDB), both within a mass error cutoff of ±5 ppm. Metabolite annotations were assigned as Metabolomics Standards Initiative (MSI) Level 3 based on accurate mass matching without MS/MS confirmation, in accordance with MSI reporting guidelines [31]. Features that could not be assigned a putative identity were classified as MSI Level 4. Pathway enrichment analysis was performed using MetaboAnalyst 6.0 based on KEGG pathway annotations, using the list of top 30 RI-associated metabolites identified from OPLS-R models. Multiple testing correction was applied using the false discovery rate (FDR), and FDR-adjusted *p*-values were calculated and reported to account for multiple testing. Overrepresentation analysis was conducted using the default background settings in MetaboAnalyst.

## 3. Results

### 3.1. Assessment of Data Quality and System Performance

**Figure 1:**
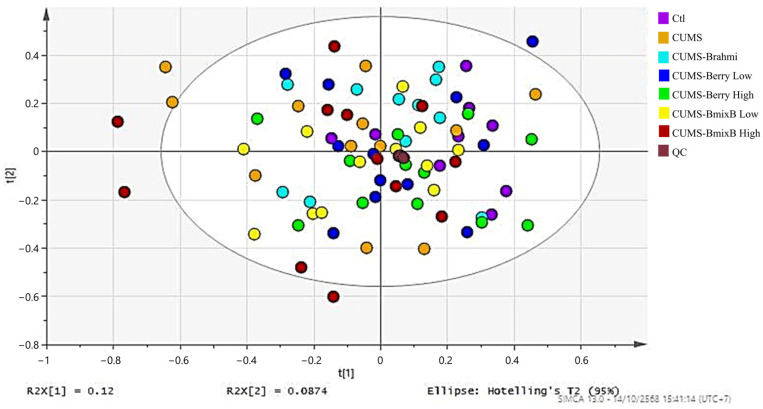
PCA score plot of all fecal metabolomics samples, including experimental groups and QC samples.

PCA was performed on all fecal metabolomics samples, including treatment groups and pooled QC samples, to evaluate data quality and overall consistency. The resulting PCA score plot (Figure 1) demonstrated that most samples were positioned within a central region. QC samples were interspersed among all treatment samples and clustered tightly, indicating stable analytical performance throughout the data acquisition.

### 3.2. Multivariate Analysis of Ctl and CUMS Groups

**Figure 2:**
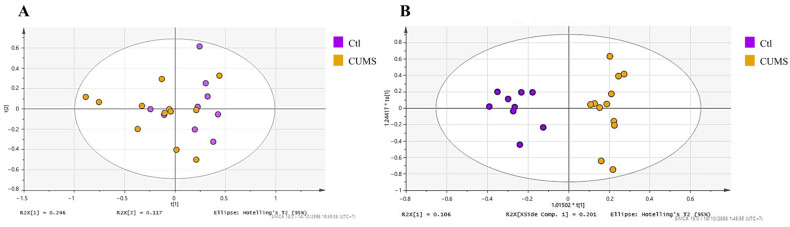
(**A**) PCA and (**B**) OPLS-DA score plots demonstrating metabolic differences and group separation between control (Ctl) and CUMS groups. * indicates the predictive component score of the OPLS model defined by the SIMCA software and does not denote statistical significance.

PCA was performed to examine global metabolic variation between the Ctl and CUMS groups. The PCA score plot (Figure 2A) showed partial separation between the groups, with some overlap remaining. Supervised OPLS-DA analysis (Figure 2B) showed separation between the groups, with model statistics of R^2^X = 0.307, R^2^Y = 0.931 and Q^2^ = 0.382, and a CV-ANOVA *p*-value of 0.0862.

### 3.3. Treatment-Specific OPLS-R Models and RI-Linked Metabolites

Pairwise OPLS-R models were generated to evaluate metabolic associations with recognition performance in each treatment condition relative to the CUMS group. The resulting score plots demonstrated covariance patterns linking metabolite intensities with behavioral outcomes, visualized as RI-gradient coloring (blue = lower RI, red = higher RI).

**Figure 3:**
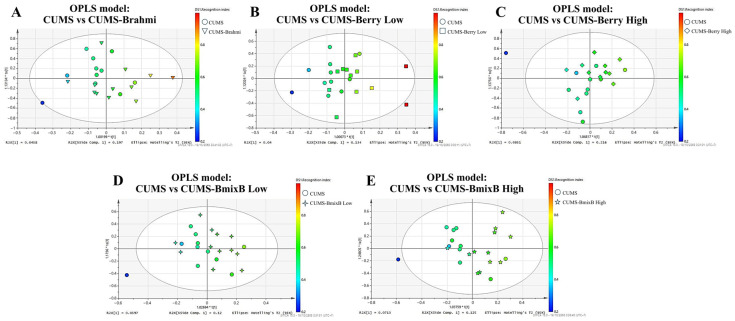
OPLS-R score plots of positive-ion mode metabolomics data illustrating metabolic separation between CUMS and treatment groups. Each model is color-graded according to RI, reflecting metabolite features associated with cognitive performance. Panels show pairwise comparisons: (**A**) CUMS vs. CUMS-Brahmi, (**B**) CUMS vs. CUMS-Berry Low, (**C**) CUMS vs. CUMS-Berry High, (**D**) CUMS vs. CUMS-BmixB Low, and (**E**) CUMS vs. CUMS-BmixB High. * indicates the predictive component score of the OPLS model defined by the SIMCA software and does not denote statistical significance.

From each model, the 30 most discriminant metabolites with VIP > 1.0 and the highest coefficient values with RI were retained and subsequently used for pathway enrichment analysis (Table 1). Model performance statistics indicated high explanatory capacity (R^2^Y = 0.823–0.997) and variable predictive strength (Q^2^ = 0.012–0.312), reflecting treatment-dependent metabolic differentiation (Table 2). Representative score plots are shown in Figure 3A–E.

Across treatment groups, distinct metabolic signatures associated with RI were observed. Unsaturated fatty acids and lipid-derived metabolites represented the most recurrent putatively annotated classes and were detected across all treatment groups. Steroid-linked metabolites appeared exclusively in the Brahmi group, whereas dipeptides were detected in both the Brahmi and Berry Low groups. Vitamin E-related metabolites were represented by alpha-tocotrienol, which was detected in the Berry Low, Berry High, and BmixB High groups. Metabolites related to tryptophan metabolism were represented by indole-3-carboxaldehyde, primarily observed in the Berry High and BmixB High groups. Bile acid-related metabolites were detected only in the BmixB Low group, as summarized in Table 1. In addition to these putatively annotated metabolites, several RI-associated features without confident metabolite identification (MSI Level 4) were also observed across treatment groups and are reported in Supplementary Table S1.

**Table 1:** OPLS-derived fecal metabolites correlated with RI in CUMS and treatment groups.

No.	Retention Time (min)	Measured *m*/*z*	Putative Identification (MSI Level 3)	Formula	Mass Error (ppm)	Adduct	Coefficient	VIP
**Key Fecal Metabolites Correlated with RI in CUMS and CUMS-Brahmi Rats.**								
1	17.3649	395.2201	Desoxycorticosterone acetate ^A^	C_23_ H_32_O_4_	0.27	[M+Na]^+^	0.3760	1.83
2	17.3702	325.2378	Decylubiquinol ^A^	C_19_ H_32_O_4_	1.20	[M+H]^+^	0.3504	1.75
3	23.3345	425.3784	Taraxasterone ^A,B^	C_30_H_48_O	0.95	[M+H]^+^	0.3420	3.78
4	16.7426	309.2429	Obtusilactone A ^A,B^	C_19_H_32_ O_3_	0.87	[M+H]^+^	0.3348	1.69
5	16.7408	381.2617	13,14-Dihydro PGF-1a ^B^	C_20_H_38_O_5_	1	[M+Na]^+^	0.3240	1.49
6	16.7411	277.2166	4,8,12,15-Octadecatetraenoic acid ^A,B^	C_18_H_28_O_2_	0.70	[M+H]^+^	0.3096	1.72
7	21.2498	626.5685	DG(20:1(11Z)/15:0/0:0) ^B^	C_38_H_72_O_5_	5	[M+NH_4_]^+^	0.2980	1.93
8	17.3698	374.2907	3-hydroxytridecanoyl carnitine ^B^	C_20_H_39_NO_5_	2	[M+H]^+^	0.2966	1.48
9	2.6122	151.1232	2,5-Dimethyl-3-propylpyrazine ^A,B^	C_9_H_14_N_2_	2.40	[M+H]^+^	0.2895	2.06
10	23.3336	465.3709	Erythrodiol ^A,B^	C_30_H_50_O_2_	0.87	[M+Na]^+^	0.2809	3.37
11	18.9849	497.3606	Camelliagenin A ^A,B^	C_30_H_50_O_4_	0.46	[M+Na]^+^	0.2635	3.22
12	20.5907	281.2479	Linoleic acid ^A,B^	C_18_H_32_O_2_	0.60	[M+H]^+^	0.2635	1.77
13	19.2534	481.3658	22alpha-Hydroxyerythrodiol ^A,B^	C_30_H_50_O_3_	0.81	[M+Na]^+^	0.2610	3.22
14	16.2847	279.2323	alpha-Linolenic acid ^A,B^	C_18_H_30_O_2_	0.51	[M+H]^+^	0.2563	1.61
15	16.2815	351.2511	Tetrahydrocorticosterone ^A,B^	C_21_H_34_O_4_	−4.69	[M+H]^+^	0.2471	1.52
16	17.3463	265.2530	(±)-(*Z*)-2-(5-Tetradecenyl)cyclobutanone ^A^	C_18_H_32_O	0.35	[M+H]^+^	0.2413	1.90
17	20.2539	297.2792	4,6-Nonadecanedione ^A,B^	C_19_H_36_O_2_	0.98	[M+H]^+^	0.2396	1.44
18	17.2297	295.2272	17-Hydroxylinolenic acid ^A,B^	C_18_H_30_O_3_	0.15	[M+H]^+^	0.2393	1.33
19	23.1057	497.3971	Heterobetulin ^B^	C_30_H_50_O_3_	1	[M+K]^+^	0.2377	3.08
20	17.227	349.2355	3beta,15beta,17alpha-Trihydroxy-pregnenone ^B^	C_21_H_32_O_4_	5	[M+H]^+^	0.2293	1.27
21	17.3459	283.2636	Oleic acid ^A,B^	C_18_H_34_O_2_	0.80	[M+H]^+^	0.2258	1.87
22	4.2593	245.1863	Leucyl-leucine ^A,B^	C_12_H_24_N_2_O_3_	1.31	[M+H]^+^	0.2151	1.39
23	14.2996	295.2272	17-Hydroxylinolenic acid ^A,B^	C_18_H_30_O_3_	0.15	[M+H]^+^	0.2022	1.42
**Key Fecal Metabolites Correlated with RI in CUMS and CUMS-Berry Low Rats.**								
24	2.6122	151.1232	2,5-Dimethyl-3-propylpyrazine ^A,B^	C_9_H_14_N_2_	2.40	[M+H]^+^	0.4854	2.91
25	14.7818	165.0912	Phenethyl acetate ^A^	C_10_H_12_O_2_	0.97	[M+H]^+^	0.3944	3.83
26	9.7265	442.3687	alpha-Tocotrienol ^A,B^	C_29_H_44_O_2_	1.93	[M+NH_4_]^+^	0.3925	2.91
27	5.5474	265.1914	Sophoranol ^A^	C_15_H_24_N_2_O_2_	1.41	[M+H]^+^	0.3300	1.73
28	2.5598	231.1706	Isoleucyl-valine ^A,B^	C_11_H_22_N_2_O_3_	0.75	[M+H]^+^	0.3239	2.33
29	21.3275	539.2989	Ganoderic acid A ^A,B^	C_30_H_44_O_7_	−1.64	[M+Na]^+^	0.3185	1.69
30	21.3286	523.3249	Frangulanine ^A,B^	C_28_H_44_N_4_O_4_	−0.28	[M+Na]^+^	0.2987	1.55
31	3.8753	361.1763	(8*S*,9*S*,10*R*,11*S*,13*S*,14*S*,17*S*)-11,17-Dihydroxy-10,13-dimethyl-17-prop-1-ynyl-9,11,12,14,15,16-hexahydro-8*H*-cyclopenta[a]phenanthren-3-one ^B^	C_22_H_26_O_3_	3	[M+Na]^+^	0.2859	2.73
32	8.9838	333.2428	Alphaxalone ^A^	C_21_H_32_O_3_	1.57	[M+H]^+^	0.2758	1.58
33	4.6503	486.2322	(3*S*)-4-[[(1*S*)-1-Carboxy-2-phenylethyl]amino]-3-[8-(diaminomethylideneamino) octanoylamino]-4-oxobutanoic acid ^B^	C_22_H_33_N_5_O_6_	0	[M+Na]^+^	0.2699	2.70
34	17.3649	395.2201	Desoxycorticosterone acetate ^A^	C_23_H_32_O_4_	0.27	[M+Na]^+^	0.2605	1.09
35	10.7767	407.2799	3-Oxocholic acid ^A,B^	C_24_H_38_O_5_	1.15	[M+H]^+^	0.2599	1.66
36	5.1928	304.1658	Tryptophyl-valine ^A,B^	C_16_H_21_N_3_O_3_	0.05	[M+H]^+^	0.2563	1.16
37	9.1331	219.1746	beta-Vetivone ^A^	C_15_H_22_O	−0.10	[M+H]^+^	0.2542	1.65
38	12.1156	288.2901	Palmitic acid methyl ester ^A^	C_17_H_34_O_2_	1.39	[M+NH_4_]^+^	0.2537	1.20
39	7.7852	430.2959	Boviquinone 4 ^A,B^	C_26_H_36_O_4_	−0.91	[M+NH_4_]^+^	0.2536	1.27
40	8.3525	407.2911	N-Eicosapentaenoyl serine ^B^	C_23_H_35_NO_4_	2	[M+NH_4_]^+^	0.2495	1.33
41	17.3702	325.2378	Decylubiquinol ^A^	C_19_H_32_O_4_	1.20	[M+H]^+^	0.2480	1.00
42	11.5363	274.2745	Palmitic acid ^A,B^	C_16_H_32_O_2_	0.56	[M+NH_4_]^+^	0.2454	1.29
43	3.1289	327.1919	Fulvine ^A^	C_16_H_23_NO_5_	−0.20	[M+NH_4_]^+^	0.2452	2.54
44	4.8668	163.1119	4-Isopropylphenylacetaldehyde ^A,B^	C_11_H_14_O	0.91	[M+H]^+^	0.2397	1.30
45	1.7237	137.0460	Hypoxanthine ^A,B^	C_5_H_4_N_4_O	4.57	[M+H]^+^	0.2390	1.28
46	17.8274	309.2429	Obtusilactone A ^A,B^	C_19_H_32_O_3_	0.87	[M+H]^+^	0.2373	1.64
**Key Fecal Metabolites Correlated with RI in CUMS and CUMS-Berry High Rats.**								
47	8.0269	193.0859	Eugenyl formate ^A,B^	C_11_H_12_O_3_	−0.11	[M+H]^+^	0.1554	2.26
48	11.9285	298.2743	Linoleic acid ^A,B^	C_18_H_32_ O_2_	1.58	[M+NH_4_]^+^	0.1274	2.23
49	12.7018	300.2903	Oleic acid ^A,B^	C_18_H_34_O_2_	1.13	[M+NH_4_]^+^	0.1274	2.54
50	7.1513	320.1528	Metconazole ^A,B^	C_17_H_22_ClN_3_O	1.59	[M+H]^+^	0.1554	2.35
51	23.3345	425.3784	Taraxasterone ^A,B^	C_30_H_48_O	0.95	[M+H]^+^	0.1265	2.36
52	12.9511	427.2252	7alpha-hydroxy-3-oxochol-4-en-24-oic Acid ^B^	C_24_H_36_O_4_	2	[M+K]^+^	0.1238	1.85
53	9.7265	442.3687	alpha-Tocotrienol ^A,B^	C_29_H_44_O_2_	1.93	[M+NH_4_]^+^	0.1211	2.70
54	19.8781	512.5046	Cer(d18:0/14:0) ^B^	C_32_H_65_NO_3_	2	[M+H]^+^	0.1185	1.71
55	12.951	799.5129	PG(20:5(6E,8Z,11Z,14Z,17Z)-OH(5)/i-17:0) ^B^	C_43_H_75_O_11_P	1	[M+H]^+^	0.1178	2.31
56	4.6999	146.0602	Indole-3-carboxaldehyde ^A,B^	C_9_H_7_NO	−0.10	[M+H]^+^	0.1029	1.80
57	8.9838	333.2428	Alphaxalone ^A,B^	C_21_H_32_O_3_	1.57	[M+H]^+^	0.1020	1.40
58	23.3336	465.3709	Erythrodiol ^A,B^	C_30_H_5_0O_2_	0.87	[M+Na]^+^	0.1014	1.61
59	19.1221	326.3057	Oleoyl ethanolamide ^A,B^	C_20_H_39_NO_2_	1.38	[M+H]^+^	0.0917	1.54
60	19.2534	481.3658	22alpha-Hydroxyerythrodiol ^A,B^	C_30_H_50_O_3_	0.81	[M+Na]^+^	0.0911	1.53
61	18.1662	439.3577	Ganoderol A ^A,B^	C_30_H_46_O_2_	0.89	[M+H]^+^	0.0908	1.57
62	10.7767	407.2799	3-Oxocholic acid ^A,B^	C_24_H_38_O_5_	1.15	[M+H]^+^	0.0904	1.31
63	18.9849	497.3606	Camelliagenin A ^A,B^	C_30_H_50_O_4_	0.46	[M+Na]^+^	0.0899	1.55
**Key Fecal Metabolites Correlated with RI in CUMS and CUMS-BmixB Low Rats.**								
64	7.1513	320.1530	Metconazole ^A^	C_17_H_22_Cl N_3_O	1.59	[M+H]^+^	0.2641	3.10
65	20.5907	281.2479	Linoleic acid ^A,B^	C_18_H_32_O_2_	0.60	[M+H]^+^	0.2376	2.28
66	17.8274	309.2427	Obtusilactone A ^A,B^	C_19_H_32_O_3_	0.87	[M+H]^+^	0.2281	2.31
67	15.3963	473.3633	Ganoderiol E ^A,B^	C_30_H_48_O_4_	1.06	[M+H]^+^	0.1992	2.63
68	11.9285	298.2745	9(*Z*),11(*E*)-Conjugated linoleic acid ^A^	C_18_H_32_O_2_	1.58	[M+NH_4_]^+^	0.1813	2.19
69	2.6122	151.1232	2,5-Dimethyl-3-propylpyrazine ^A,B^	C_9_H_14_N_2_	2.40	[M+H]^+^	0.1792	2.01
70	12.7018	300.2902	Oleic acid ^A,B^	C_18_H_34_O_2_	1.13	[M+NH_4_]^+^	0.1777	2.73
71	17.6039	324.2901	Linoleoyl ethanolamide ^A,B^	C_20_H_37_NO_2_	0.73	[M+H]^+^	0.1767	1.96
72	12.1818	297.2429	12-Hydroxy-8,10-octadecadienoic acid ^A,B^	C_18_H_32_O_3_	0.54	[M+H]^+^	0.1754	2.52
73	19.1221	326.3057	Oleoyl ethanolamide ^A,B^	C_20_H_39_NO_2_	1.38	[M+H]^+^	0.1735	1.99
74	20.2539	297.2792	4,6-Nonadecanedione ^A,B^	C_19_H_36_O_2_	0.98	[M+H]^+^	0.1607	1.41
75	4.4959	493.2817	Erinacine P ^A,B^	C_27_H_40_O_8_	3.38	[M+H]^+^	0.1491	1.66
76	16.6587	319.2249	12-Hydroxy-8,10-octadecadienoic acid ^A,B^	C_18_H_32_O_3_	0.54	[M+Na]^+^	0.1445	2.07
77	15.6312	431.2776	Cholic acid ^A,B^	C_24_H_40_O_5_	0.10	[M+Na]^+^	0.1445	1.45
78	12.9511	427.2252	7alpha-hydroxy-3-oxochol-4-en-24-oic acid ^B^	C_24_H_36_O_4_	2	[M+K]^+^	0.1433	1.66
79	12.951	799.5129	PG(20:5(6E,8Z,11Z,14Z,17Z)-OH(5)/i-17:0) ^B^	C_43_H_75_O_11_P	1	[M+H]^+^	0.1421	1.60
80	17.8214	372.3842	Tricosanoic acid ^A,B^	C_23_H_46_O_2_	−1.47	[M+NH_4_]^+^	0.1380	1.62
81	10.561	256.2639	Palmitic amide ^A,B^	C_16_H_33_NO	0.82	[M+H]^+^	0.1362	1.63
82	23.3345	425.3784	Taraxasterone ^A,B^	C_30_H_48_O	0.95	[M+H]^+^	0.1315	1.26
83	7.7852	430.2959	Boviquinone 4 ^A,B^	C_26_H_36_O_4_	−0.91	[M+NH_4_]^+^	0.1283	1.20
**Key Fecal Metabolites Correlated with RI in CUMS and CUMS-BmixB High Rats.**								
84	12.7018	300.2902	Oleic acid ^A,B^	C_18_H_34_O_2_	1.13	[M+NH_4_]^+^	0.2198	3.04
85	19.8781	512.5046	Cer(d18:0/14:0) ^B^	C_32_H_65_NO_3_	2	[M+H]^+^	0.1965	2.85
86	9.7265	442.3687	alpha-Tocotrienol ^A,B^	C_29_H_44_O_2_	1.93	[M+H]^+^	0.1953	1.89
87	15.3963	473.3633	Ganoderiol E ^A,B^	C_30_H_48_O_4_	1.06	[M+H]^+^	0.1914	2.55
88	12.1297	318.3008	3*R*-hydroxy-octadecanoic acid ^B^	C_18_H_36_O_3_	2	[M+NH_4_]^+^	0.1718	2.62
89	2.6122	151.1232	2,5-Dimethyl-3-propylpyrazine ^A,B^	C_9_H_14_N_2_	2.40	[M+H]^+^	0.1683	1.51
90	20.8967	540.5360	Tetrahydro-6-(2-hydroxy-16,19-dimethylhexacosyl)-4-methyl-2H-pyran-2-one ^A,B^	C_34_H_66_O_3_	1.21	[M+NH_4_]^+^	0.1682	2.71
91	15.6177	377.3532	5-Decanoyl-2-nonylpyridine ^A,B^	C_24_H_41_NO	1.27	[M+NH_4_]^+^	0.1660	2.15
92	7.5428	257.0811	Isoliquiritigenin ^A,B^	C_15_H_12_O_4_	1.03	[M+H]^+^	0.1608	2.30
93	14.9102	363.3376	Macamide B ^A,B^	C_23_H_39_NO	0.82	[M+NH_4_]^+^	0.1521	1.80
94	14.8737	291.1960	(9*Z*,11*E*,13*E*,15*Z*)-4-Oxo-9,11,13,15-octadecatetraenoic acid ^A,B^	C_18_H_26_O_3_	1.19	[M+H]^+^	0.1514	2.31
95	1.8499	211.1444	L,L-Cyclo(leucylprolyl) ^A,B^	C_11_H_18_N_2_O_2_	1.31	[M+H]^+^	0.1482	2.49
96	23.3345	425.3784	Taraxasterone ^A,B^	C_30_H_48_O	0.95	[M+H]^+^	0.1473	1.60
97	1.3707	197.1288	Fasoracetam ^A,B^	C_10_H_16_N_2_O_2_	−1.15	[M+H]^+^	0.1446	2.41
98	8.9838	333.2428	Alphaxalone ^A^	C_21_H_32_O_3_	1.57	[M+H]^+^	0.1422	1.67
99	17.4054	384.3842	(*E*)-2-Tetracosenoic acid ^A,B^	C_24_H_46_O_2_	−0.60	[M+NH_4_]^+^	0.1412	2.70
100	10.883	316.2850	5-Hexyltetrahydro-2-furanoctanoic acid ^A,B^	C_18_H_34_O_3_	1.05	[M+NH_4_]^+^	0.1342	2.23
101	8.0269	193.0861	Eugenyl formate ^A^	C_11_H_12_O_3_	−0.11	[M+H]^+^	0.1286	1.40
102	12.3797	401.3381	Erucoylacetone ^B^	C_25_H_46_O_2_	2	[M+Na]^+^	0.1284	1.36
103	10.561	256.2639	Palmitic amide ^A,B^	C_16_H_33_NO	0.82	[M+H]^+^	0.1249	1.93
104	14.042	349.3219	Docosane ^B^	C_22_H_46_	3	[M+K]^+^	0.1174	1.82

Databases used for putative identification: ^A^ = METLIN; ^B^ = HMDB.

**Table 2:** The parameters of the OPLS-R model.

Group	R^2^X	R^2^Y	Q^2^	CV-ANOVA
CUMS vs. CUMS-Brahmi	0.455	0.991	0.278	0.8089
CUMS vs. CUMS-Berry Low	0.527	0.997	0.012	1.0000
CUMS vs. CUMS-Berry High	0.301	0.823	0.312	0.1310
CUMS vs. CUMS-BmixB Low	0.319	0.925	0.103	0.9248
CUMS vs. CUMS-BmixB High	0.197	0.894	0.089	0.7789

### 3.4. Pathway Enrichment Analysis

**Figure 4:**
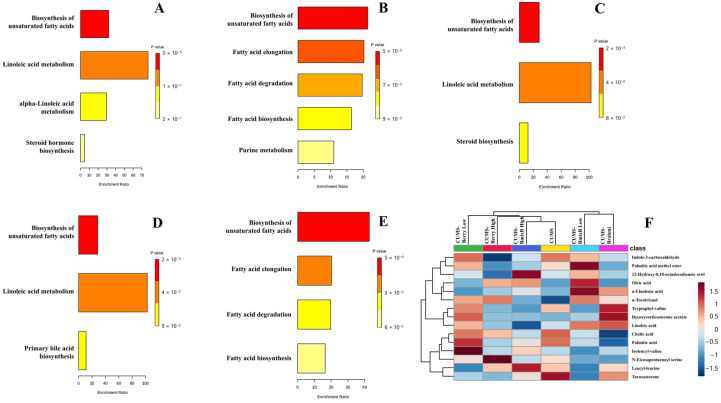
Summary plots of overrepresentation analysis (ORS) showing enriched metabolic pathways between CUMS and treatment groups: (**A**) Brahmi, (**B**) Berry-Low, (**C**) Berry-High, (**D**) BmixB-Low, and (**E**) BmixB-High. Panel (**F**) presents a heatmap of discriminant RI-associated fecal metabolites across groups, illustrating treatment-dependent metabolic regulation consistent with cognitive improvement patterns.

Pathway enrichment analysis indicated treatment-specific metabolic modulation associated with RI, with four pathways showing enrichment across the five intervention groups: biosynthesis of unsaturated fatty acids, linoleic acid metabolism, alpha-linolenic acid metabolism, and fatty acid elongation (Figure 4A–E).

In the CUMS-Brahmi group, three pathways showed enrichment, including biosynthesis of unsaturated fatty acids (*p* < 0.0001), linoleic acid metabolism (*p* = 0.0130), and alpha-linolenic acid metabolism (*p* = 0.0334). Metabolites contributing to these pathways included linoleic acid (C_18_H_32_O_2_; *m*/*z* 281.2479; RT 20.59 min), α-linolenic acid (C_18_H_30_O_2_; *m*/*z* 279.2323; RT 16.28 min), and oleic acid (C_18_H_34_O_2_; *m*/*z* 283.2636; RT 17.35 min).

In the CUMS-Berry Low group, enrichment was observed for biosynthesis of unsaturated fatty acids (*p* = 0.0463) and fatty acid elongation (*p* = 0.0488). Palmitic acid (C_16_H_32_O_2_; *m*/*z* 274.2742; RT 11.54 min) was identified as the principal metabolite contributing to these enriched pathways.

For the CUMS-Berry High group, enrichment was detected in biosynthesis of unsaturated fatty acids (*p* = 0.0016) and linoleic acid metabolism (*p* = 0.0097). Linoleic acid (C_18_H_32_O_2_; *m*/*z* 298.2743; RT 11.93 min) and oleic acid (C_18_H_34_O_2_; *m*/*z* 300.2903; RT 12.70 min) were identified as key associated metabolites.

A similar enrichment pattern was observed in the CUMS-BmixB Low group, in which biosynthesis of unsaturated fatty acids (*p* = 0.0016) and linoleic acid metabolism (*p* = 0.0097) were enriched. Corresponding RI-linked metabolites included linoleic acid (C_18_H_32_O_2_; *m*/*z* 281.2479; RT 20.59 min) and oleic acid (C_18_H_34_O_2_; *m*/*z* 300.2903; RT 12.70 min).

In the CUMS-BmixB High group, enrichment was observed for biosynthesis of unsaturated fatty acids (*p* = 0.0005) and fatty acid elongation (*p* = 0.0488). Oleic acid (C_18_H_34_O_2_; *m*/*z* 300.2902; RT 12.70 min) and a palmitic acid-mapped feature (putatively annotated as palmitic amide) represented the principal RI-associated metabolites contributing to these pathway enrichments.

Across treatment groups, biosynthesis of unsaturated fatty acids was the only pathway consistently enriched across all interventions. In contrast, the remaining pathways showed treatment- and dose-dependent variation. Linoleic acid metabolism was enriched in the Brahmi, Berry High, and BmixB Low, whereas alpha-linolenic acid metabolism appeared only in the Brahmi group. Fatty acid elongation was enriched in the Berry Low and BmixB High groups, suggesting elongation-driven lipid remodeling at these dose levels. Dose-related divergence was most apparent in the mixed Thai berry and BmixB interventions, in which low- and high-dose treatments displayed different pathway memberships despite partially overlapping profiles. Brahmi demonstrated the broadest pathway engagement (3 pathways), whereas the remaining treatments modulated 2 pathways each. FDR-adjusted *p*-values for pathway enrichment are provided in Table S2.

## 4. Discussion

This study integrated untargeted fecal metabolomics with behavioral assessment to explore metabolic signatures associated with the cognitive effects of Brahmi, mixed Thai berry, and their combined extracts under chronic stress. Although the supervised OPLS models exhibited high R^2^Y values, the low to modest Q^2^ values indicate limited predictive performance. Consistent with this observation, the CV-ANOVA results (Table 2) support interpretation of these models within an exploratory framework rather than for prediction. Cross-validation plots (Supplementary Figures S1 and S2) further illustrate the constrained predictive ability of the supervised models. As highlighted by Szymańska et al. (2011), supervised models with constrained predictive strength may still be appropriate for exploratory metabolomics [32]. Accordingly, the OPLS-based results in the present study are interpreted within this exploratory framework.

Across treatment groups, a similar metabolic pattern was observed, with most RI-associated metabolites belonging to lipid-related classes, particularly unsaturated fatty acids and steroid-linked metabolites. This aligns with evidence indicating that lipid metabolism may support neuronal membrane stability, synaptic signaling, and cognitive regulation under stress [33]. Although this shared lipid signature was consistent across interventions, distinct metabolic features were also evident. Brahmi showed unique associations with alpha-linolenic-linked pathways, consistent with reports suggesting that Brahmi may influence neuroprotective processes, including modulation of neurotransmission, inflammatory signaling, and cellular stress responses, which may intersect with lipid remodeling mechanisms relevant to cognition under chronic stress [34]. In contrast, mixed Thai berry extract exhibited a dose-dependent metabolic shift consistent with hormetic responses to dietary polyphenols, where the lower dose yielded more favorable behavioral outcomes than the higher dose, consistent with prior reports of biphasic effects of polyphenol-rich interventions [35]. The BmixB group displayed a distinct metabolic response, with the high-dose treatment associated with the greatest improvement in recognition performance. Both doses were characterized by enrichment of unsaturated fatty acid biosynthesis; however, secondary metabolic patterns differed, with linoleic acid metabolism more evident at the low dose and fatty-acid elongation pathways more prominent at the high dose.

Overall, the results indicate that lipid-related pathways are involved across all interventions; however, the metabolic response differs depending on the treatment and dose. Such patterns imply that restoration of lipid homeostasis, together with treatment-dependent engagement of neuroactive lipid pathways, may contribute to cognitive improvement. The following sections examine these treatment-specific metabolic signatures in greater detail, discussing their biological relevance in the context of existing evidence.

### 4.1. Metabolic Signatures Associated with Cognitive Improvement in the CUMS-Brahmi Group

Most RI-associated metabolites in the Brahmi group were lipid-derived, particularly unsaturated fatty acids, including oleic acid, linoleic acid, and alpha-linolenic acid. Pathway enrichment analysis indicated enrichment of pathways related to unsaturated fatty acid biosynthesis, linoleic acid metabolism, and alpha-linoleic acid metabolism. These polyunsaturated fatty acids have been implicated in neuronal membrane structure, synaptic signaling, and neuroinflammatory regulation [33]. Their increased abundance may reflect compensatory responses to stress-induced lipid dysregulation, potentially involving enhanced polyunsaturated fatty acid turnover and metabolic availability. Linoleic acid and alpha-linolenic acid also act as precursors of bioactive lipid mediators that modulate corticosteroid and immune signaling [36]. Together, these findings suggest that recognition performance may be associated with modulation of lipid metabolic pathways under chronic stress.

Although the steroid hormone biosynthesis pathway was not statistically significant in enrichment analysis, desoxycorticosterone acetate and tetrahydrocorticosterone were up-regulated in the CUMS-Brahmi group. Chronic stress has been reported to suppress neurosteroid formation and dysregulate the HPA axis; therefore, even partial elevation of steroid-related features may indicate a compensatory response associated with stress adaptation [37]. This trend aligns with previous evidence suggesting that Brahmi may modulate neurosteroid signaling and support GABAergic function, which may contribute to anxiolytic and neuroprotective outcomes [34].

A number of metabolites, although not mapped to known metabolic pathways, may indicate additional metabolic processes potentially associated with Brahmi extract. Leucyl-leucine, a gut microbiota-derived dipeptide, was also elevated in the Brahmi group. Although direct associations between this metabolite and cognitive function have not been established, dipeptides of microbial origin have been proposed as indicators of gut metabolic activity. Such gut-derived metabolites are known to participate in microbiota-driven signaling processes relevant to stress regulation and host behavioral outcomes [38].

### 4.2. Metabolic Signatures Associated with Cognitive Improvement in the CUMS-Berry Low Group

Pathway enrichment analysis (Figure 4B) indicated enrichment of unsaturated fatty acid biosynthesis and fatty acid elongation, with palmitic acid emerging as the primary metabolite associated with these pathway-level changes. Its moderate elevation in the CUMS-Berry Low group may reflect altered lipid turnover and mitochondrial fatty acid utilization, processes previously linked to neuronal lipid signaling and memory-associated metabolic adaptation [39]. This interpretation aligns with reports showing that low-dose polyphenol exposure and berry-derived anthocyanins can modulate lipid metabolism, promote β-oxidation, and attenuate lipid-derived inflammatory signaling [40].

Moreover, detection of N-eicosapentaenoyl serine, a conjugate of eicosapentaenoic acid, suggests possible involvement of endogenous omega-3-derived lipid mediator pathways associated with anti-inflammatory signaling [41]. Several non-lipid compounds also exhibited notable correlations with RI. alpha-Tocotrienol, a vitamin E isomer with antioxidant activity, was positively associated with recognition performance. Tocotrienols have been reported to protect neuronal membranes from lipid peroxidation and to modulate neuroinflammatory signaling, processes that may be associated with cognitive function under oxidative stress [42]. Two dipeptides, isoleucyl-valine and tryptophyl-valine, also showed positive correlations with RI. Tryptophan-containing dipeptides may be associated with serotonergic and kynurenine metabolic pathways, which are central nodes in gut–brain axis regulation [43]. Such modulation could provide a potential mechanism through which mixed Thai berry extract may contribute to stress resilience and cognitive function.

Notably, the observation that metabolic modulation occurred even at the low anthocyanin dose used in the CUMS-Berry Low group is consistent with previous rodent findings. Kim and Oh (2013) demonstrated that anthocyanin exposure equivalent to 0.043 mg C3GE/kg BW produced measurable cognitive improvements [29], indicating that anthocyanins can exert biological activity at this dose range. This supports the plausibility of detecting metabolomic signatures at low intake levels and is consistent with prior evidence that anthocyanin activity can occur without high doses.

### 4.3. Metabolic Signatures Associated with Cognitive Improvement in the CUMS-Berry High Group

In the CUMS-Berry High group, pathway enrichment indicated involvement of unsaturated fatty acid biosynthesis, similar to the pattern observed in the CUMS-Berry Low group. However, unlike the low-dose condition, steroid biosynthesis emerged as a non-significant enrichment pattern, suggesting partially altered metabolic patterns at higher extract exposure.

Despite similarities in metabolic patterns, the RI in CUMS-Berry High rats was lower than in CUMS-Berry Low rats, consistent with a dose-dependent biphasic effect rather than a linear improvement. Interestingly, Indole-3-carboxaldehyde was detected among the top 30 features. This microbial tryptophan derivative is recognized as an endogenous aryl hydrocarbon receptor (AhR) ligand and has been implicated in mucosal immune signaling and gut barrier processes [44,45], suggesting continued involvement of gut microbial signaling pathways in the CUMS-Berry High group.

### 4.4. Metabolic Signatures Associated with Cognitive Improvement in the CUMS-BmixB Low Group

The metabolic response in the CUMS-BmixB Low group partially overlapped with the profiles observed in the individual extract treatments. Unsaturated fatty acid biosynthesis was significantly enriched, whereas linoleic acid metabolism also showed enrichment, consistent with patterns seen in both Brahmi and mixed Thai berry-treated groups. Linoleic and oleic acids were among the RI-associated metabolites, suggesting that modulation of membrane lipid balance and unsaturated fatty acid turnover may contribute to improved recognition performance [36].

Unlike the single-extract interventions, bile acid-related metabolites were uniquely detected in this treatment group. The emergence of primary bile acid biosynthesis as a trending pathway is noteworthy. Metabolites such as cholic acid and 7alpha-hydroxy-3-oxochol-4-en-24-oic acid showed positive coefficients, suggesting partial involvement of steroidogenic and lipid detoxification processes. This metabolic pattern aligns with stress-responsive neuroendocrine regulation, as bile acid intermediates can function as signaling molecules acting through FXR and TGR5 receptors within the gut–brain axis [46,47]. Their presence may therefore reflect a potential microbial or hepatic response associated with the combined extract, rather than being attributable to either component alone.

### 4.5. Metabolic Signatures Associated with Cognitive Improvement in the CUMS-BmixB High Group

In the CUMS-BmixB High group, the enriched pathways remained centered on lipid metabolism, with biosynthesis of unsaturated fatty acids and fatty acid elongation identified as the main pathways associated with metabolic modulation. However, the pathway pattern differed, since linoleic acid metabolism and the bile acid-related pathway observed in the low-dose condition were not retained at the high dose. Instead, the CUMS-BmixB High group showed additional involvement of fatty acid degradation and fatty acid biosynthesis, which may reflect an increased emphasis on lipid turnover and remodeling [36,39].

Although some RI-associated metabolites were shared between the two doses, the overall response at the higher dose reflected a different metabolic pattern rather than a dose-dependent extension of the low-dose profile. The behavioral improvement observed in the CUMS-BmixB High group was consistent with this pathway shift, suggesting that increased extract exposure may be associated with differences in lipid-related metabolic patterns. Overall, these findings indicate that the metabolic response to the combined extract is dose sensitive, resulting in a profile distinct from that observed at the lower dose.

## 5. Conclusions

This study applied untargeted fecal metabolomics to investigate metabolic pathways associated with the cognitive effects of Brahmi, mixed Thai berry, and their combined extracts in a CUMS model. Both behavioral and metabolomic outcomes demonstrated that all interventions were associated with metabolic pathways related to recognition memory, with biosynthesis of unsaturated fatty acids emerging as the only pathway consistently represented across treatment groups. Distinct metabolic signatures differentiated the treatment groups. In the Brahmi group, changes were characterized by metabolites linked to linoleic and alpha-linolenic acid metabolism. For the mixed Thai berry extract, metabolic alterations varied by dose, with lipid modulation observed in both doses, while amino acid-derived metabolites appeared specifically in the low-dose group. Only the low-dose combined extract showed association with bile acid-related metabolites. When considered alongside the behavioral outcomes, these patterns suggest that recognition performance may be associated with dose-dependent adjustments in lipid metabolic processes and interactions involving gut-derived metabolites under chronic stress conditions.

Overall, the findings suggest that fecal metabolomics may help identify metabolic signatures associated with memory performance in response to single and combined herbal extracts. These exploratory associations offer biochemical context relevant to potential cognitive effects. Further studies incorporating targeted metabolite validation, microbiome functional profiling, and tissue-specific analyses are needed to confirm these pathways and to clarify their potential mechanistic contributions within the gut–brain axis.

## 6. Limitations

This study has several methodological limitations that should be acknowledged. First, most metabolite annotations were based on MS^1^ data and database matching without MS/MS confirmation, corresponding to MSI Level 3; targeted MS/MS validation is therefore required to strengthen compound identification in future studies. Second, only male rats were used, and potential sex-dependent differences in metabolic responses and stress susceptibility were not evaluated. Because sex hormones can modulate lipid and bile acid metabolism, future studies should include female cohorts to determine whether the metabolic signatures observed here are sex-specific or broadly applicable. Third, microbiome sequencing was not performed alongside metabolomics, limiting the ability to directly link bacterial taxa with metabolite alterations. Fourth, the use of SpeedVac drying at 40 °C may influence sample composition and partially affect thermolabile metabolites, such as short-chain fatty acids and conjugated polyphenols; lyophilization may better preserve heat-sensitive compounds and should be considered in future work. Additionally, the supervised multivariate models may be susceptible to overfitting, and the predictive results should be interpreted with caution. Future studies incorporating permutation testing, larger sample sizes, or independent validation sets will be important for strengthening model robustness. Moreover, as this untargeted fecal metabolomics study identifies associations rather than causal mechanisms, future mechanistic validation will be required to determine whether the observed metabolic changes directly influence cognition outcomes. Together, these limitations temper the conclusions and indicate clear directions for future research.

## Data Availability

The original contributions presented in this study are included in the article/Supplementary Materials. Further inquiries can be directed to the corresponding authors.

## Supplementary Materials

The following supporting information can be downloaded at: https://www.mdpi.com/article/10.3390/antiox15010056/s1, Table S1: Unidentified fecal metabolites correlated with RI in CUMS and treatment groups. Table S2: Pathway enrichment analysis with raw and FDR-adjusted *p*-values. Figure S1: Cross-validation plot of the OPLS-DA model comparing Control (Ctl) vs. CUMS. Figure S2: Cross-validation plots of OPLS-R models comparing CUMS and treatment groups.
