# Supplementary material for: Fecal Metabolomic Insights into Memory-Associated Pathways Modulated by Bacopa monnieri, Mixed Thai Berry, and Combined Extracts in Rats Under Chronic Unpredictable Mild Stress
Source: Antioxidants (Basel). 2026 Jan 1;15(1):56. doi: 10.3390/antiox15010056 (PMC12837653; doi:10.3390/antiox15010056)
Supplement: Supplementary file 1 [file antioxidants-15-00056-s001.zip › antioxidants-4031801-supplementary.pdf]

Supplementary Materials

Table S1 reports retention time, measured *m/z*, coefficient, and VIP values for each feature derived from the OPLS-R models. Features are grouped according to treatment comparisons (CUMS-Brahmi, CUMS-Berry Low, CUMS-Berry High, CUMS-BmixB Low, and CUMS-BmixB High) to illustrate treatment-specific unidentified metabolic signals associated with improved recognition performance.

Inclusion of these unidentified features provides transparency regarding RI-associated metabolic patterns and highlights potential metabolites of interest for future structural elucidation and targeted analysis.

Table S1. Unidentified fecal metabolites correlated with RI in CUMS and treatment groups.

No.	Retention time (min)	Measured *m/z*	Unknown metabolites (MSI Level 4)	Coefficient	VIP
**Key fecal metabolites correlated with RI in CUMS and CUMS-Brahmi rats.**					
1	6.8162	229.1413	Unidentified	0.3942	2.52
2	13.4781	470.2600	Unidentified	0.3860	2.40
3	5.5077	652.4102	Unidentified	0.2932	2.47
4	20.4736	497.3398	Unidentified	0.2878	3.27
5	23.3353	481.3448	Unidentified	0.2777	3.38
6	20.5341	515.4073	Unidentified	0.2723	1.52
7	20.0136	498.364	Unidentified	0.2553	3.21
**Key fecal metabolites correlated with RI in CUMS and CUMS-Berry Low rats.**					
8	6.8162	229.1413	Unidentified	0.4975	2.08
9	21.2157	626.5686	Unidentified	0.3206	1.95
10	3.3379	144.0446	Unidentified	0.3077	1.90
11	4.4558	485.2289	Unidentified	0.2909	2.88
12	7.5638	554.3697	Unidentified	0.2903	2.10
13	11.1796	336.2619	Unidentified	0.2862	1.28
14	10.1537	815.5604	Unidentified	0.2549	2.33
**Key fecal metabolites correlated with RI in CUMS and CUMS-Berry High rats.**					
15	6.0348	288.1267	Unidentified	0.1354	1.95
16	6.0275	306.1373	Unidentified	0.1265	2.38
17	12.9506	372.262	Unidentified	0.1354	2.20
18	11.7286	392.2883	Unidentified	0.1274	2.80
19	15.2336	413.3018	Unidentified	0.1274	2.47
20	15.2345	432.3157	Unidentified	0.1227	1.99
21	20.0136	498.3640	Unidentified	0.1210	3.01
22	21.2157	626.5686	Unidentified	0.1184	2.17
23	21.2498	626.5685	Unidentified	0.1080	2.09
24	20.404	653.5358	Unidentified	0.1007	1.42
25	12.9499	390.2725	Unidentified	0.1004	1.04
26	11.1796	336.2619	Unidentified	0.0969	1.70
27	18.9558	423.3628	Unidentified	0.0929	1.56

No.	Retention time (min)	Measured *m/z*	Unknown metabolites (MSI Level 4)	Coefficient	VIP
**Key fecal metabolites correlated with RI in CUMS and CUMS-BmixB Low rats.**					
28	6.0275	306.1373	Unidentified	0.2661	2.92
29	12.8899	445.2129	Unidentified	0.1853	2.28
30	4.5198	389.2399	Unidentified	0.1746	1.90
31	6.0348	288.1267	Unidentified	0.1704	1.96
32	12.9499	390.2725	Unidentified	0.1690	1.95
33	4.4558	485.2289	Unidentified	0.1664	1.81
34	20.5902	335.2561	Unidentified	0.1598	1.66
35	16.6584	280.2358	Unidentified	0.1554	1.99
36	1.4023	230.2231	Unidentified	0.1516	1.53
37	15.6221	416.3073	Unidentified	0.1502	1.51
**Key fecal metabolites correlated with RI in CUMS and CUMS-BmixB High rats.**					
38	20.0136	498.364	Unidentified	0.1772	1.71
39	19.3178	572.4536	Unidentified	0.1453	2.40
40	22.9957	652.5843	Unidentified	0.1393	2.25
41	11.1796	336.2619	Unidentified	0.1284	1.50
42	7.1289	393.2543	Unidentified	0.1246	1.11
43	7.5638	554.3697	Unidentified	0.1211	2.40
44	16.8792	442.3770	Unidentified	0.1207	2.42
45	15.2345	432.3157	Unidentified	0.1196	1.31
46	4.5198	389.2399	Unidentified	0.1193	1.22

Table S2 presents pathway enrichment results for each comparison between the CUMS group and the corresponding treatment groups (CUMS-Brahmi, CUMS-Berry Low, CUMS-Berry High, CUMS-BmixB Low, and CUMS-BmixB High). Both raw and FDR-adjusted p-values are provided to ensure transparent interpretation of pathway enrichment results and to distinguish exploratory signals from those remaining significant after correction for multiple testing.

Table S2. Pathway enrichment analysis with raw and FDR-adjusted p-values

Pathway	P-value	FDR-adjusted p-value
**CUMS vs CUMS-Brahmi**		
Biosynthesis of unsaturated fatty acids	4.63×10^{-5}	0.0037
Linoleic acid metabolism	0.0129	0.5180
alpha-Linoleic acid metabolism	0.0334	0.8910
Steroid hormone biosynthesis	0.208	1.0000
**CUMS vs CUMS-Berry Low**		
Biosynthesis of unsaturated fatty acids	0.0463	1.0000
Fatty acid elongation	0.0488	1.0000
Fatty acid degradation	0.0501	1.0000
Fatty acid biosynthesis	0.0602	1.0000
Purine metabolism	0.1100	1.0000

Pathway	P-value	FDR-adjusted p-value
**CUMS vs CUMS-Berry High**		
Biosynthesis of unsaturated fatty acids	0.0016	0.1260
Linoleic acid metabolism	0.0097	0.3890
Steroid biosynthesis	0.0779	1.0000
**CUMS vs CUMS-BmixB Low**		
Biosynthesis of unsaturated fatty acids	0.0016	0.1260
Linoleic acid metabolism	0.0097	0.3890
Primary bile acid biosynthesis	0.0871	1.0000
**CUMS vs CUMS- BmixB High**		
Biosynthesis of unsaturated fatty acids	5.32×10^{-4}	0.0426
Fatty acid elongation	0.0488	1.0000
Fatty acid degradation	0.0501	1.0000
Fatty acid biosynthesis	0.0602	1.0000

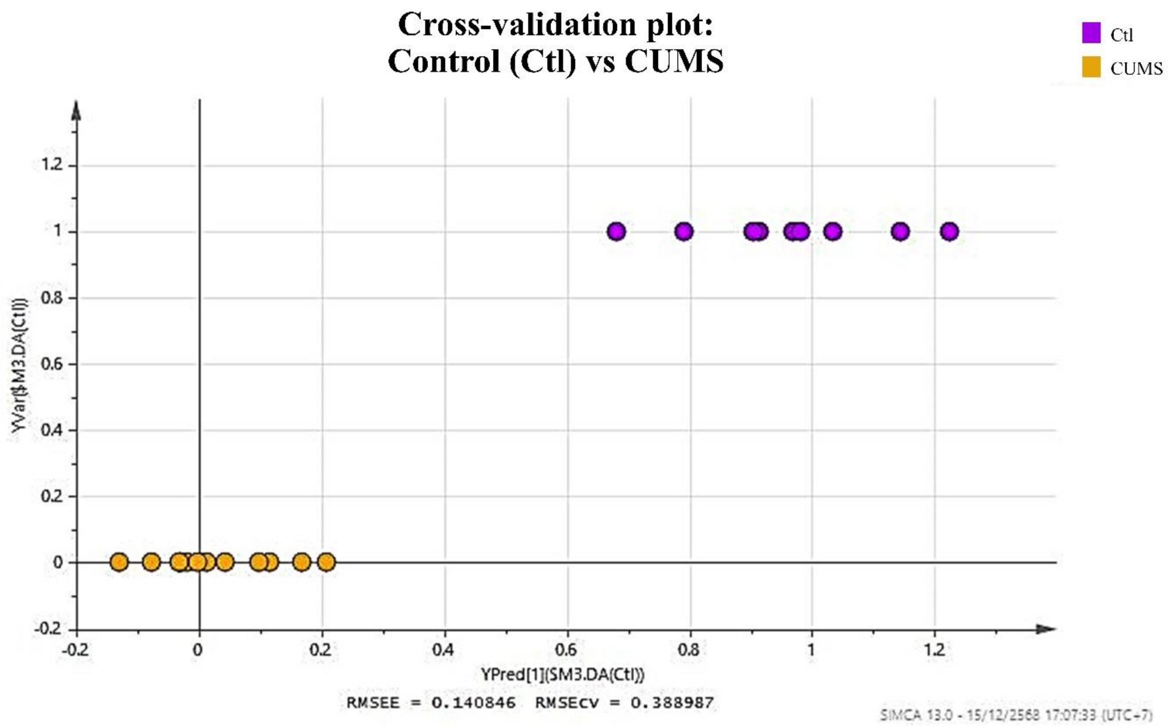

Figure S1. Cross-validation plot of the OPLS-DA model comparing Control (Ctl) vs CUMS

Cross-validation in Figure S1 was performed to examine the supervised OPLS-DA model used to distinguish metabolic profiles between the Ctl and CUMS groups. The cross-validation plot illustrates changes in predictive performance (Q^2) across model complexity and is provided to support evaluation of the supervised discrimination between unstressed and stressed conditions within an exploratory metabolomics framework.

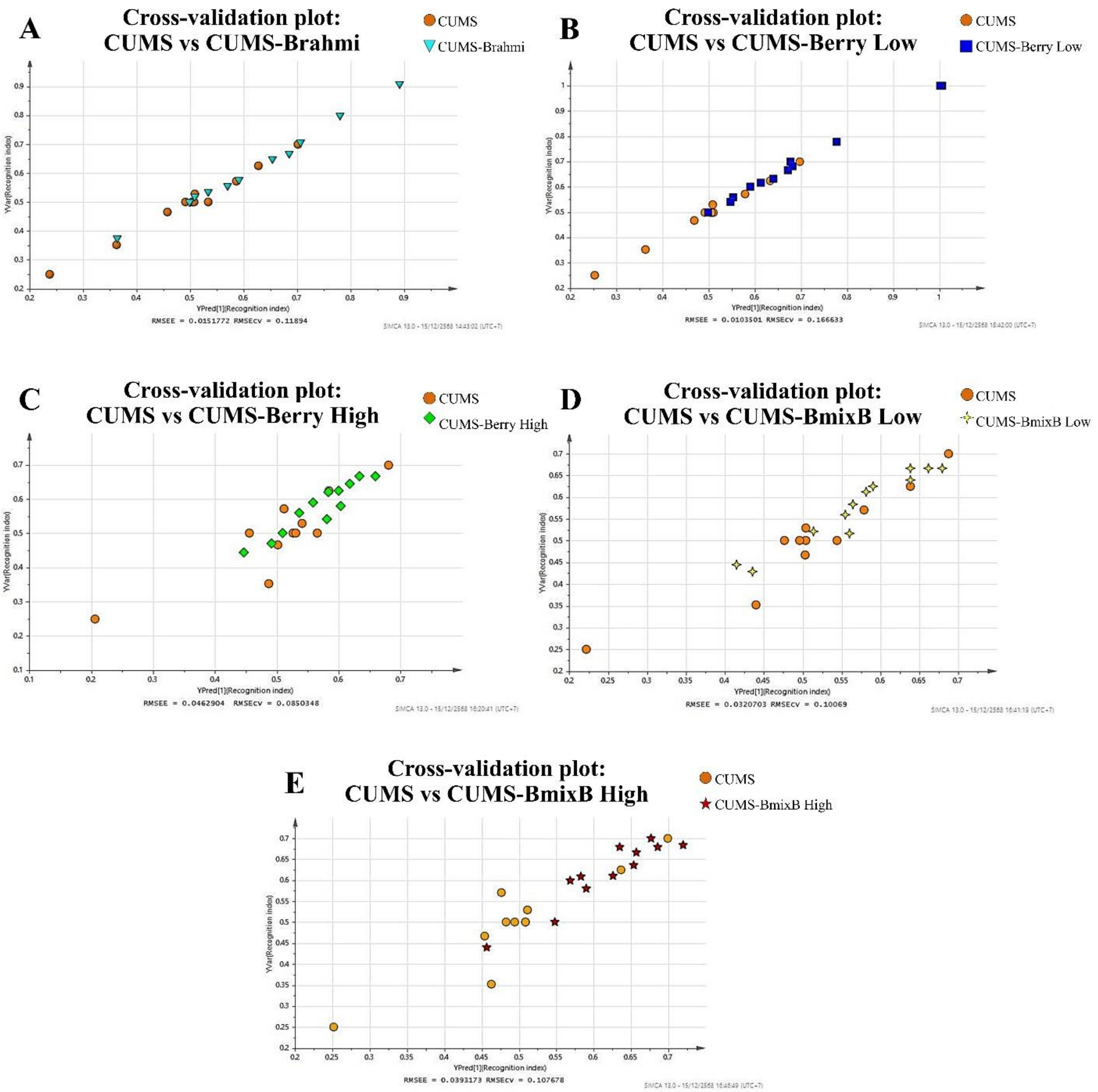

Figure S2. Cross-validation plots of OPLS-R models comparing CUMS and treatment groups

Cross-validation in Figure S2 was conducted for multiple OPLS-R models comparing the CUMS group with each treatment group. The cross-validation plots illustrate model behavior and variation in predictive performance (Q^2) across treatment conditions, supporting interpretation of metabolic features associated with RI under different intervention settings.
